# Evidence That Cigarette Smoking Alters Alveolar Type I Cell Nuclear Fractal Dimension

**DOI:** 10.7759/cureus.50254

**Published:** 2023-12-10

**Authors:** Sotirios Maipas, Periklis G Foukas, Ioannis G Panayiotides, Ioannis Vamvakaris, Nikolaos Kavantzas

**Affiliations:** 1 First Department of Pathology, School of Medicine, National and Kapodistrian University of Athens, Athens, GRC; 2 Second Department of Pathology, School of Medicine, “Attikon” University Hospital, National and Kapodistrian University of Athens, Athens, GRC; 3 Department of Pathology, “Sotiria” Athens Hospital, Athens, GRC; 4 First Department of Pathology, School of Medicine, National and Kapodistrian University of Athens, Athens General Hospital "Laikon", Athens, GRC

**Keywords:** smoking, lung, fractal dimension, box-counting dimension, alveolar type i cells

## Abstract

A large number of alveolar type I and II cells from the lungs of both smokers and non-smokers was collected using 40x magnification histological images from our digital archive. These images underwent a transformation into binary images of nuclear contours, followed by the application of the box-counting method. Statistical analysis revealed a significant difference in the mean box-counting dimension values between type I cells of smokers and non-smokers. However, no significant difference was observed in the mean fractal dimensions of alveolar type II cells. This study provides preliminary evidence of the impact of cigarette smoking on the nuclear shape of alveolar type I cells. Given the high toxicity of cigarette smoke to lung cells and the interconnection between morphology and function, further study is needed to understand its impact on the nuclear shape of these cells. Future research should also explore the effects of second-hand smoke on cell shape.

## Editorial

The box-counting dimension is a popular fractal dimension that relies on the sequential covering of objects of interest with square boxes of varying sizes. It is usually equated to the slope of the regression line on a log-log plot, which correlates the scale (defined as the ratio of box size to image size) with the number of square boxes needed to cover the object. This calculation can be performed, as in our case, on nuclear contours using the FracLac plug-in in the ImageJ application (National Institutes of Health, Bethesda, Maryland, United States). In addition to the box-counting dimension, FracLac also provides a goodness-of-fit value for each calculation (i.e., the r-squared value, indicating how well the regression line fits the set of measurements) [1]. The diagnostic potential of the goodness-of-fit index was demonstrated in a previous study we conducted using breast, normal, benign, and malignant, cells [2]. The current study employs the same method for calculating the box-counting dimension as the one used in the study of breast cells.

In particular, we created nuclear contours using Wolfram Mathematica 10.4 (Wolfram Research, Inc., Champaign, Illinois, United States). Using 40x magnification histological images from our digital archive, we collected alveolar type I and type II cells from the lungs of smokers and non-smokers. Specifically, we analyzed 231 alveolar type I cells (114 from smokers and 117 from non-smokers) and 240 alveolar type II cells (118 from smokers and 122 from non-smokers). The statistical analysis was conducted using the Data Analysis tool of Microsoft Excel 365 (Microsoft Corporation, Redmond, Washington, United States). The research protocol was approved by the School of Medicine of the National and Kapodistrian University of Athens, Greece.

**Figure 1:**
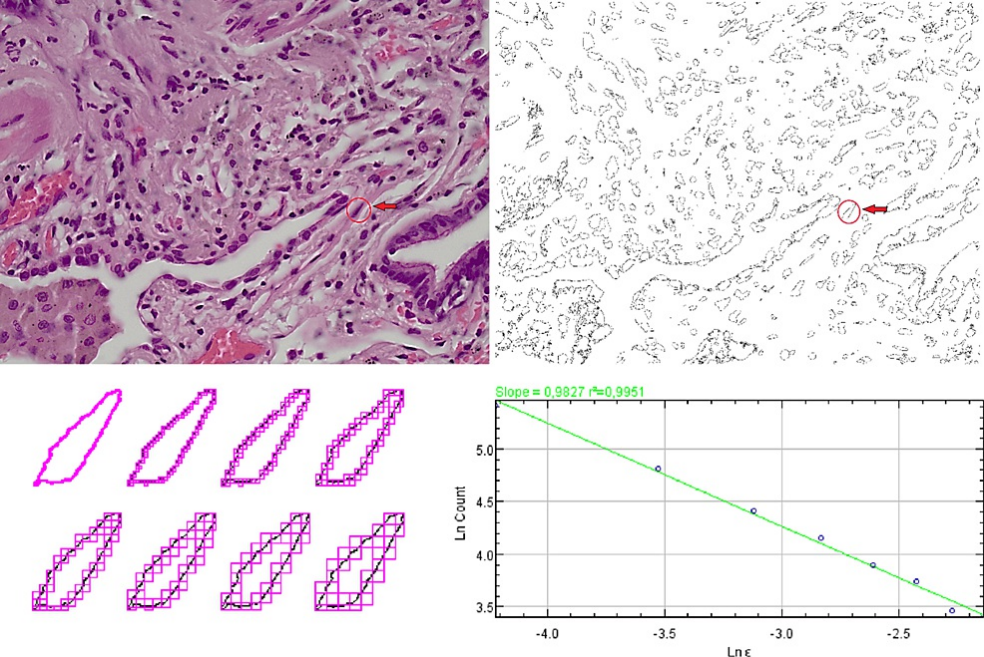
A brief description of the box-counting method using a random alveolar type I cell

The following image (Figure 1) gives a brief overview of the methodology described above.

The mean fractal dimension of alveolar type I cells for smokers was 0.99980 (variance: 0.00105, min: 0.9325, max: 1.0928), and for non-smokers, it was 1.03752 (variance: 0.00243, min: 0.9453, max: 1.1917). A t-test for unequal variances indicated a statistically significant difference between the mean values of these two groups (p-value < 0.01). In terms of the goodness-of-fit, the mean r-squared value was 0.99567 for smokers and 0.99348 for non-smokers. The same test revealed a statistically significant difference between the mean values of the goodness-of-fit indices (p-value < 0.01). In contrast, no statistically significant difference was found between the mean values of fractal dimensions, and of goodness-of-fit indices, in alveolar type II cells.

Alveolar type I cells are highly susceptible to oxidant injury induced by cigarette smoking, and they also provide a large surface area where damage can manifest [3]. This study provides preliminary evidence on the impact of cigarette smoking on the nuclear shape of alveolar type I cells. We observed a notable difference in the mean fractal dimension values of these cells between smokers and non-smokers. This difference likely reflects the characteristic effects of smoking on alveolar type I lung cells. Moreover, the generally lower susceptibility of alveolar type II cells to oxidant injury and to cigarette smoke [3] may explain the lack of statistically significant difference in the mean fractal dimensions of type II cells.

Given the high toxicity of cigarette smoke to alveolar cells [4], and the interconnection between morphology and function [5], the impact on the nuclear shape of alveolar type I cells needs further study. Future research should also focus on the effects of second-hand smoke on cell shape, on the role of the intensity of both active and passive smoking, and on identifying potential changes in nuclear shape that may occur after smoking cessation. Indeed, we strongly believe that the outcome of this study necessitates larger-scale research that includes smokers, non-smokers often exposed to second-hand smoke, and non-smokers without a history of passive smoking. It must be mentioned here that for the cells examined in this study, no information regarding passive smoking was available.
